# Supplementary material for: Patient’s Perspective of Telemedicine in Poland—A Two-Year Pandemic Picture
Source: Int J Environ Res Public Health. 2022 Dec 22;20(1):115. doi: 10.3390/ijerph20010115 (PMC9819744; doi:10.3390/ijerph20010115)
Supplement: Supplementary file 1 [file ijerph-20-00115-s001.zip › ijerph-2036580-supplementary.pdf]

Abbreviation	Definition
**CAWI**	Computer Assisted Web Interview
**COVID-19**	Coronavirus Disease
**GP**	General Practicioner
**ICT**	Information Communications Technology
**IQS**	International Quality Service
**NHF**	The National Health Fund
**OFBOR**	Polish Association of Public Opinion and Market Research Firms
**PKJPA**	Programme for Quality Control of Interviewers' Work
**ROF**	'Rise or fall? Short and long-term health and psychosocial trajectories of COVID-19 pandemics'

Table S1. Used abbreviation

SZ.1. Did you have a tele-counseling or e-visit during the COVID-19 pandemic?

		1. yes	2. no	If SZ1_1 and SZ1_2 answer. 1 then SZ1a SZ1a. In the course of a teleconsultation or e-visit, did the doctor see you through the camera?			
				Yes, always 4	In half of the cases 3	Occasionally 2	Never 1
1	With your GP	◻	◻	◻	◻	◻	◻
2	With specialist	◻	◻	◻	◻	◻	◻
3	I wanted to get but I was unable to make an appointment	◻	◻				

Table S2. Use of telemedicine by responders

SZ.5. Below are other people's opinions on teleconsultation or e-visits during the COVID-19 pandemic. Please indicate how much you agree or disagree with each of the opinions?

		0 (I definitely do not agree)	1	2	3	4	5	6	7	8	9	10 (Definitely agree)	Not applicable
1	Are ineffective	◻	◻	◻	◻	◻	◻	◻	◻	◻	◻	◻	◻
2	They save my time: e.g. I can easily obtain a prescription, get a referral	◻	◻	◻	◻	◻	◻	◻	◻	◻	◻	◻	◻
3	It happened that the doctor had no insight into my test results	◻	◻	◻	◻	◻	◻	◻	◻	◻	◻	◻	◻
4	I felt unprotected	◻	◻	◻	◻	◻	◻	◻	◻	◻	◻	◻	◻
5	I am concerned that due to teleconsultations or e-visits, I will not be diagnosed in time with a serious illness in me or my relatives	◻	◻	◻	◻	◻	◻	◻	◻	◻	◻	◻	◻
6	Considering the COVID-19 pandemic, I believe a system of teleconsultations and e-visits is appropriate	◻	◻	◻	◻	◻	◻	◻	◻	◻	◻	◻	◻
7	It is very difficult to receive teleconsultation or e-visit when I or my child need it the most	◻	◻	◻	◻	◻	◻	◻	◻	◻	◻	◻	◻

Table S3. Respondents' opinions on teleconsultation
